# Satisfaction with healthcare services in South Africa: results of the national 2010 General Household Survey

**DOI:** 10.11604/pamj.2014.18.172.4084

**Published:** 2014-06-22

**Authors:** Kathryn H Jacobsen, Takahiro Hasumi

**Affiliations:** 1 Department of Global and Community Health, George Mason University, USA

**Keywords:** Patient satisfaction, health services accessibility, healthcare disparities, public hospitals, private hospitals, South Africa

## Abstract

**Introduction:**

The 1998 and 2003 Demographic and Health Surveys suggested increasing rates of dissatisfaction with health services in South Africa. The goal of this analysis was to examine national healthcare satisfaction rates in 2010.

**Methods:**

We conducted weighted logistic regression analysis of data from 22,959 household representatives who participated in the nationally-representative 2010 General Household Survey (GHS).

**Results:**

In total, 88.5% of participants were somewhat or very satisfied with their last visit to their usual healthcare provider, including 84.6% of those visiting a public provider and 97.3% of those consulting a private provider. Satisfaction rates were lower for black South Africans (87.0%) and low income households (86.3% of households with monthly incomes less than 2500 rands) than for white South Africans (96.0%) and high income households (94.0% of those with monthly incomes of at least 8000 rands) (p<0.001). However, after adjusting for provider type, there were few differences in satisfaction rates by race/ethnicity and income level.

**Conclusion:**

The analysis suggests that differences in satisfaction with healthcare services in South Africa by racial/ethnic group and income level are due in large part to different rates of use of private providers.

## Introduction

The strategic plan released in 2010 by South Africa's national Department of Health includes “improved patient care and satisfaction” as one of 20 key outcomes for the 2009-2014 Medium Term Strategic Framework (MTSF) time period [1]. The plan calls for the establishment of a national customer care program and ombuds office to investigate and resolve complaints, and requires public hospitals to begin conducting annual satisfaction surveys [1]. This policy is designed, in part, to address negative perceptions toward the healthcare system that were highlighted by the 1998 and 2003 South Africa Demographic and Health Surveys (DHS). Between 1998 and 2003, reported dissatisfaction rates among those using health services within the past 30 days rose from 12% to 23% of patients at public hospitals, 12% to 22% at public clinics (day hospitals), 7% to 12% at private hospitals, 6% to 8% for private doctors, 4% to 8% for chemists (pharmacists), and 3% to 13% for dentists [2].

This study provides a more current set of satisfaction and dissatisfaction rates by analyzing a nationally-representative sample of South Africans surveyed in 2010. The specific objectives of this analysis are (1) to estimate the proportion of adult South Africans who report being satisfied with their most recent visit to their usual healthcare provider, (2) to compare the satisfaction rates of South Africans by population group (race/ethnicity) and income, and (3) to identify the factors associated with being satisfied with healthcare services.

## Methods

The General Household Survey (GHS), a nationally-representative cross-sectional survey of private households and workers' hostels, has been conducted by Statistics South Africa (SSA) annually since 2002 [3]. A total of 25,548 households containing 95,918 individuals consented to participate in the 2010 GHS, which was conducted between July and September through home visits by trained interviewers. Questions about use of and satisfaction with the healthcare system were asked at the household level. All required ethics reviews and approvals were acquired prior to implementation of the study.

The GHS uses a two-stage, stratified sampling design [3]. First, primary sampling units (PSUs) are randomly selected from across the country. PSUs consist of 100 to 500 households (called dwelling units, or DUs), and are based on the enumeration areas from the 2001 national census. After the DUs within the sampled PSUs are stratified by several socio-demographic characteristics, DUs are sampled from each PSU using a randomized probability proportional to size (RPPS) function that generates a national sample of DUs that matches key characteristics of the national census. For example, in the final sampling frame the proportion of metropolitan and non-metropolitan households and the proportion of households by province match the national proportions. In total, 93.4% of sampled DUs participated in the 2010 GHS.

The household survey instrument contained a series of questions about the household's interaction with the healthcare system. The primary question of interest for this paper asked *“How satisfied were you (the respondent) with the service you received during (your last) visit to the health facility normally used by the household? ”* The analysis in this paper is restricted to the 22,959 households for which the household representative who was interviewed provided an answer to this question. Responses of “very satisfied” or “somewhat satisfied” were classified as satisfaction for this analysis.

The question *“If anyone in this household gets ill and decides to seek medical help, where do most of them usually go first?”* was used to ascertain whether the household generally uses public clinics and hospitals or private sector providers. Public facilities provide free care for most medical conditions, while private facilities usually charge a service fee [4, 5]. Only 6.6% of the 2010 GHS participants whose last visit to a healthcare facility was to a public provider reported paying for the visit, while 93.4% of those who went to a private provider paid for the visit out of pocket or via a medical aid scheme (health insurance).

Additional key variables included the race/ethnicity of the head of household and the monthly household income. The four racial/ethnic population groups were listed in the questionnaire as Black African, White, Coloured (mixed race / Cape Malay), and Indian / Asian. The monthly household income (in South African rands) included salaries, grants, business income, remittances, and pensions.

The data were weighted for analysis using household weights assigned by Statistics South Africa (SSA) to adjust for differences between the participating households and the national population. Chi-square tests and multiple logistic regression models were used to compare rates of healthcare service satisfaction for different socio-demographic groups and healthcare provider types. All tests were conducted in SPSS version 21 (IBM, New York, USA), using two-sided p-values and a significance level of α = 0.05. All mentions of statistical significance in the paper refer to test results with p<0.05.

## Results

**Figure 1:**
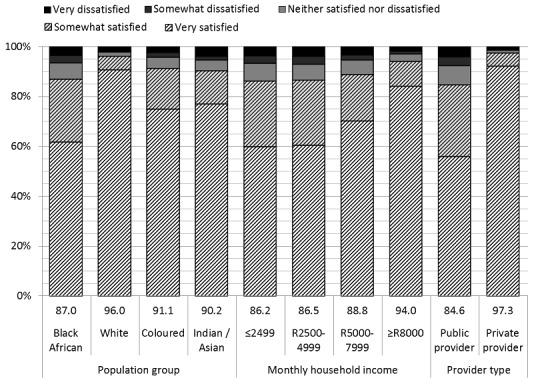
Level of satisfaction with the most recent visit to the usual healthcare provider, by the race of the head of household, monthly household income, and type of provider. The diagonal shading indicates satisfaction

Overall, 88.5% of the respondents reported being satisfied with their last visit to their usual healthcare provider. The reported satisfaction rate exceeded 80% for all population groups, all four monthly household income groups, and both public and private providers, but there were significant differences in the satisfaction levels for these groups (Figure 1).

In total, 18.6% of respondents reported using private healthcare providers and 81.4% reported using public providers. The gap in satisfaction between those attending different types of providers was substantial, with 97.3% of those visiting private providers reporting being somewhat or very satisfied with the last visit compared to 84.6% of those who visited a public provider (p<0.001) (Table 1). Dissatisfaction rates were 6% and 8% for public hospitals and public clinics, respectively, compared to 2% for private hospitals and 1% for private doctors.

**Table 1:** Proportion of participants who were somewhat or very satisfied with the most recent visit to the usual healthcare provider by population group (race/ethnicity), monthly household income, and provider type (public or private)

Provider Type	Income	Black African		White		Coloured		Asian / Indian		Total	
		n	% sat.	n	% sat.	n	% sat.	n	% sat.	n	%
Public	≤ 2499	9089	85.2	63	84.1	520	88.3	84	81.0	9756	85.3
	R2500-4999	3086	83.6	29	89.7	330	88.8	51	82.4	3496	84.2
	R5000-7999	912	82.3	29	82.8	156	87.2	22	72.7	1119	82.8
	≥ R8000	831	82.1	97	83.5	139	83.5	33	72.7	1100	82.1
	Total	14,378	84.5	254	87.0	1185	87.0	208	80.3	16,026	84.6
Private	≤ 2499	733	96.5	123	93.5	76	97.4	35	94.3	967	96.1
	R2500-4999	579	98.3	113	92.9	74	94.6	24	100.0	790	97.2
	R5000-7999	487	97.1	189	98.4	104	95.2	29	96.6	809	97.2
	≥ R8000	1309	98.5	1557	97.3	407	99.3	213	93.4	3486	97.8
	Total	3290	97.8	2485	96.9	743	97.7	386	95.6	6904	97.3
Total	≤ 2499	9822	86.0	186	90.3	596	89.4	119	84.9	10,723	86.3
	R2500-4999	3665	85.9	142	92.3	404	89.9	75	88.0	4286	86.6
	R5000-7999	1399	87.5	218	96.3	260	90.4	51	86.3	1928	88.8
	≥ R8000	2140	92.1	1654	96.5	546	95.2	246	90.7	4586	94.0
	Total	17,668	87.0	2744	96.0	1933	91.1	594	90.2	22,959	88.5

White South Africans and those from higher income households were more likely than others to report being satisfied with their last healthcare visit (p<0.001). Regression analysis suggested that these differences were primarily related to differential use of private providers: after adjusting for the usual type of provider (public vs. private), there were few differences in satisfaction rates (and even in rates of being “very satisfied”) between racial/ethnic groups or by monthly household income (Table 2).

**Table 2:** Odds ratios and 95% confidence intervals for satisfaction with the last visit to the usual healthcare provider, based on logistic regression models adjusting for type of provider, population group, and/or monthly household income

	Model 1	Model 2	Model 3	Model 4	Model 5
Dependent variable	Somewhat or very satisfied	Somewhat or very satisfied	Somewhat or very satisfied	Somewhat or very satisfied	Very satisfied
Independent variables	Provider	Provider + Population Group	Provider + Income	All	All
Private (vs. public)	**6.58** **(5.65, 7.67)**	**6.82** **(5.73, 8.12)**	**6.92** **(5.87, 8.18)**	**7.11** **(5.93, 8.53)**	**8.16** **(7.31, 9.12)**
White (vs. black)	--	0.94 (0.75, 1.18)	--	0.96 (0.76, 1.21)	**1.38** **(1.18, 1.62)**
Coloured (vs. black)	--	**1.21** **(1.02, 1.43)**	--	**1.24** **(1.05, 1.46)**	**1.42** **(1.26, 1.59)**
Indian / Asian (vs. black)	--	**0.69** **(0.52, 0.93)**	--	**0.71** **(0.53, 0.94)**	0.90 (0.72, 1.11)
≥ R8000 (vs. ≤ 2499)	--	--	0.89 (0.77, 1.03)	0.89 (0.77, 1.04)	1.00 (0.90, 1.12)
R5000-7999 (vs. ≤ 2499)	--	--	**0.84** **(0.72, 0.98)**	**0.83** **(0.71, 0.97**)	0.90 (0.81, 1.01)
R2500-4999 (vs. ≤ 2499)	--	--	0.92 (0.83, 1.03)	0.92 (0.83, 1.02)	**0.87** **(0.81, 0.94)**

Bold text indicates statistically significant odds ratios (p<0.05).

After adjustment for provider type, population group, and income, reported satisfaction with the most recent healthcare visit was somewhat higher among those with small household sizes, those without children living in the home, those with a male head of household, and those in non-urban areas (Table 3-A). Those who had not seen the usual healthcare provider in the past year reported slightly higher satisfaction rates than those with a more recent visit, but after adjustment there was no difference in satisfaction based on participation in a medical scheme (that is, a medical insurance plan) or on the selection of the provider nearest to the home rather than a more distant provider. Participants who rated their water service or electrical service as “average” or “poor” were significantly less likely than those who rated these services as “good” to say that they were satisfied with their last healthcare visit. As expected, those who had not experienced a problem such as a long wait time, unavailability of needed drugs, or rude staff during the last visit to the usual healthcare provider were much more likely to report satisfaction with their visit (Table 3-B).

**Table 3-A:** Characteristics associated with being very or somewhat satisfied with the most healthcare visit

Variable	% of households with the characteristic	% with characteristic who were satisfied	% without characteristic who were satisfied	Unadjusted OR (95% CI)	Adjusted OR† (95% CI)
Household size ≤ 3 (vs. ≥ 4)	55.3	90.0	86.5	**1.41** **(1.30, 1.54)**	**1.23** **(1.14, 1.35)**
No children (≤17 years old) in household (vs. ≥ 1 child)	43.0	90.6	86.8	**1.47** **(1.35, 1.59)**	**1.23** **(1.14, 1.35)**
Older adults (≥60 years old) in household (vs. 0 older adults)	23.3	88.6	88.4	1.01 (0.92, 1.12)	1.04 (0.94, 1.15)
Male head of household (vs. female)	61.4	89.6	86.6	**1.33** **(1.34, 1.45)**	**1.14** **(1.05, 1.23)**
Urban residential location (vs. non-urban)	65.4	89.0	87.4	**1.17** **(1.08, 1.27)**	**0.83** **(0.76, 0.90)**
More than 1 year since most recent visit to usual provider (vs. ≤ 1 year)	9.8	88.6	86.9	**1.18** **(1.03, 1.35)**	**1.16** **(1.02, 1.33)**
Medical aid scheme participation (vs. No participation)	24.8	94.7	86.4	**2.79** **(2.46, 3.15)**	1.01 (0.87, 1.19)
Usual provider is not the nearest of its kind (clinic, hospital, etc.) to home (vs. Usual provider is nearest to home)	90.8	93.2	88.0	**1.89** **(1.58, 2.25)**	1.05 (0.87, 1.26)
Rating of municipal water services (by the 84.5% of households with drinking water supplied by a municipality) as good (vs. average or poor)	63.2	91.2	84.0	**1.99** **(1.82, 2.17)**	**1.71** **(1.56, 1.88)**
Rating of electricity supply services (by the 82.6% of households that reported having a connection to the MAINS electricity supply) as good (vs. average or poor)	67.5	91.7	82.9	**2.28** **(2.08, 2.50)**	**2.16** **(1.97, 2.38)**

† Adjusted for provider type (public / private), population group, and income.

Bold text indicates statistically significant odds ratios (p<0.05).

**Table 3-B:** Characteristics associated with being very or somewhat satisfied with the most healthcare visit

Variable	% of households with the characteristic	% with characteristic who were satisfied	% without characteristic who were satisfied	Unadjusted OR (95% CI)	Adjusted OR† (95% CI)
Did not experience concerns during the last visit to the usual healthcare provider (vs. ≥1 concern)	56.1	98.2	76.1	**16.9** **(14.7, 19.3)**	**13.3** **(11.6, 15.3)**
Did not have to wait a long time during the last visit to the usual healthcare provider (vs. Long wait time)	65.1	96.1	74.2	**8.6** **(7.9, 9.5)**	**6.6** **(6.0, 7.3)**
Needed drugs were available during the last visit to the usual healthcare provider (vs. Drugs not available)	85.9	93.7	56.5	**11.5** **(10.5, 12.7)**	**9.0** **(8.2, 9.9)**
Staff were not rude or uncaring and did not turn patient away during the last visit to the usual healthcare provider (vs. Staff was rude)	89.8	93.4	45.1	**17.2** **(15.6, 18.9)**	**13.2** **(12.0, 14.6)**
The opening times were convenient during the last visit to the usual healthcare provider (vs. Time not convenient)	93.2	90.2	64.4	**5.1** **(4.6, 5.7)**	**4.3** **(3.8, 4.8)**
The cost of care was not too expensive during the last visit to the usual healthcare provider (vs. Too expensive)	95.9	88.4	90.5	0.8 (0.6, 1.0)	**2.3** **(1.8, 3.0)**
The facilities were clean during the last visit to the usual healthcare provider (vs. Not clean)	96.3	89.7	56.5	**6.7** **(5.8, 7.8)**	**6.0** **(5.1, 6.9)**

† Adjusted for provider type (public / private), population group, and income.

Bold text indicates statistically significant odds ratios (p<0.05).

## Discussion

We found relatively small differences in satisfaction rates for different population (racial/ethnic) groups after adjustment for provider type, even though significant disparities exist between various population groups within South Africa in terms of overall (not provider-adjusted) healthcare satisfaction rates as well as mortality and morbidity rates, life expectancies, and access to healthcare services and the social determinants of health [6–8]. Similarly, our analysis showed relatively small differences in satisfaction for different income levels after adjusting for provider type, even though significant health disparities exist by income quintile within South Africa [9]. In other words, there were similar levels of satisfaction among those attending private providers regardless of race/ethnicity and income, and similar but lower levels of satisfaction for those attending public providers regardless of race/ethnicity and income.

Consistent with most patient satisfaction surveys [10], the vast majority of participants in the 2010 GHS reported being satisfied with the service provided during their last visit to their usual healthcare provider. When comparing the 2010 GHS results to previous DHS studies in South Africa, satisfaction appears to be increasing and dissatisfaction decreasing [2]. However, the use of different questionnaire items and study designs makes a direct comparison impossible [11]. The 2010 GHS results also suggest reduced disparities in satisfaction with healthcare services since the nationwide 1998 Kaiser Household Survey, which found that, after adjusting for provider type, white South Africans and those with high socioeconomic status (SES) were about 1.5 times more likely to report “excellent” service than black South Africans and lower SES households [12]. The statistically insignificant or weak adjusted odds ratios for the 2010 GHS suggest that disparities in service level by race/ethnicity and income may have been reduced in the intervening years, even though inequalities by provider type remain.

The significant differences in overall satisfaction with healthcare services that continue to exist by race/ethnicity and income may largely be attributable to differences in ability to access private healthcare services. Those who visit private providers remain significantly more likely than those going to public providers to be satisfied with their last clinical encounter. This is a continuing trend, since the 1998 and 2003 DHS studies also found that those receiving health services from public providers were less satisfied with their care than those who attended private providers [2, 13].

It is important to note that satisfaction rates alone cannot be considered evidence of high quality care. Patients' impressions of the quality of their care may be based primarily on good interpersonal communication with their clinicians, and patients may have poor ability to rate their doctors' technical skills [14]. Additionally, patients' ratings of healthcare consultations are dependent on their expectations for the visit [15, 16]. It is possible that the lower satisfaction rates at public facilities in the 2010 South Africa GHS are due to the perception, whether valid or not, that public facilities offer lower-quality services than private facilities. Patients expecting a negative experience may be more likely than those with higher expectations to report having unsatisfying encounters with the healthcare system. However, it is also possible that low expectations for the quality of services provided at public healthcare facilities may result in higher levels of reported satisfaction for those services than would be assigned for services of equal perceived quality provided by a private practitioner for whom performance expectations were higher.

Additionally, participants' responses about healthcare service satisfaction may be influenced by their education levels, culture, and other factors. For example, consider the positive association between ratings of the water and electrical systems and reported satisfaction with the most recent healthcare visit. It is possible (and perhaps likely) that areas with unreliable utilities also offer lower quality healthcare services, thereby leading to consistently low ratings of both utilities and health services. However, it is also possible that some survey participants have a preference for providing neutral answers like “average” or “neither satisfied or dissatisfied” to questionnaire items, while others have a preference for providing affirmative answers during interviews [17]. The possibility of various forms of bias associated with survey questions that ask about attitudes and perceptions requires careful interpretation of analytic results.

## Conclusion

Despite the known limitations of population-based client satisfaction surveys, these studies often yield helpful information about areas that could be targeted for improvement [18], such as the quality and availability of staff, the condition of the facilities, the availability of medications and equipment, and the cost of services, among other factors [2, 13, 19–23]. A poor performance in any of these areas may lead a patient to feel dissatisfied with his or her visit, yet healthcare providers may not be aware of the problems that reduce patient perceptions of care at their own facilities because healthcare workers' attitudes and perceptions have been shown to differ from those of patients [11]. Actively seeking to understand and address the concerns of public and private health facility patients and their families may help to improve healthcare access, health services, health status, and healthcare satisfaction in South Africa.
